# Minimally invasive endoscopic treatment for unicameral bone cysts: technique and outcomes in a series of 33 pediatric and adolescent patients

**DOI:** 10.1186/s13018-025-06557-7

**Published:** 2025-12-18

**Authors:** Lenian Zhou, Zengxin Jiang, Zeng Zhang, Qingcheng Yang, Ting Yuan

**Affiliations:** Department of Orthopaedics, Shanghai Sixth People’s Hospital Affiliated to Shanghai Jiao Tong University School of Medicine, Shanghai, People’s Republic of China

**Keywords:** Adolescent, Bone tumor, Endoscopic, Minimally invasive, Pediatric, Unicameral bone cysts

## Abstract

**Objective:**

To evaluate the clinical efficacy and safety of a minimally invasive endoscopic protocol—combining curettage, sclerotherapy, and injectable bone grafting—in the treatment of unicameral bone cysts (UBCs).

**Methods:**

From March 2019 to August 2024, 33 pediatric and adolescent patients with UBCs were treated using a standardized minimally invasive endoscopic curettage combined with sclerotherapy and injectable bone grafting at our institution. The cohort included 24 males and 9 females, with a mean age of 10.1 years (range 4–20 years). Patients were followed for an average of 48.4 months (range 12–76 months). Radiographic healing rates, functional score, and complications were assessed.

**Results:**

According to the modified Neer classification, complete or partial radiographic healing was achieved in 30 cases (90.9%), while 3 cases experienced persistent or recurrent cyst (9.1%). Almost all patients recovered excellent function. No postoperative pathological fractures or growth plate disturbances were observed during a mean follow-up of 48.4 months. Complications were limited to one episode of transient pain and one case of mild swelling, both self-limiting without intervention. Postoperative hospitalization was brief (mean, 1.2 days), and cosmetic outcomes were excellent, with minimal portal scars, far less conspicuous than those from traditional open procedures.

**Conclusion:**

Endoscopic minimally invasive surgery combining curettage, sclerotherapy, and injectable bone grafting appears to be a safe and effective option for the management of UBCs in children and adolescents, achieving high healing rates and low complication risks. Nevertheless, given the single-arm design and limited sample size, larger controlled studies with long-term follow-up are needed to confirm these findings.

**Supplementary Information:**

The online version contains supplementary material available at 10.1186/s13018-025-06557-7.

## Introduction

Unicameral bone cysts (UBCs) are benign, fluid-filled intraosseous lesions predominantly affecting the metaphysis of long bones in children and adolescents [1, 2]. While typically asymptomatic, their presence can weaken the bone cortex, making them prone to pathological fractures [3]. The exact pathogenesis of UBCs remains debated, but theories suggest abnormal fluid dynamics, prostaglandin imbalances, and osteoblastic activity within the cyst cavity play a role [4]. Common locations include the humerus and femur, although they can occur in various bones, including the calcaneus and ilium [1].

Historically, treatment options for UBCs have varied widely, and multiple treatments have been proposed ranging from conservative observation to open curettage, while no consensus has been reached on the optimal treatment [5, 6]. Percutaneous steroid injection therapy emerged as the first minimally invasive treatment option, demonstrating success rates between 42–60% in various series [7–9]. While representing an improvement over observation, these results remained suboptimal, particularly for larger cysts [7]. Open curettage with bone grafting subsequently offered more predictable outcomes than injection therapy. However, this approach carries significant limitations, including extensive soft tissue dissection, carry risks of physeal damage in growing children, and they typically necessitate prolonged recovery periods [7]. Elastic stable intramedullary fixation/nailing (ESIF/ESIN) has been reported as an effective treatment for UBCs [10]. In addition to providing mechanical stability, it may promote cyst decompression and aeration, facilitating healing [11]. However, it often requires secondary implant removal and is more suitable for patients with pathological fractures [12].

Recent advancements in orthopedic and bone tumor surgery have shifted towards minimally invasive techniques to reduce surgical morbidity while maintaining efficacy [13, 14]. Advances in endoscopic techniques and sclerosing agents have created new therapeutic opportunities for UBC management. Endoscopic curettage offers several theoretical advantages including direct visualization of the cyst cavity for thorough evacuation, preservation of periosteal blood supply, and reduced soft tissue trauma [15, 16]. Polidocanol sclerotherapy has demonstrated efficacy in reducing recurrence, and artificial bone grafts (such as calcium phosphate cements) provide mechanical support and promote healing [17, 18]. This study integrates these strategies to optimize UBC treatment.

In this study, a comprehensive evaluation of endoscopic management of UBCs with adjuvant sclerotherapy and injectable bone grafting in children and adolescents was reported. The aim was to assess radiographic healing rates, functional score, and complications.

## Methods

This prospective cohort study was conducted at a tertiary bone tumor center following approval by the institutional review board. Patients under 20 years of age with radiologically and clinically confirmed UBCs were considered for inclusion. Symptomatic lesions or those deemed at high fracture risk were selected for surgical intervention. Symptomatic lesions were defined as those producing persistent or activity-related pain affecting daily function or sports. High fracture risk was defined as cysts occupying > 50% of the bone diameter, cortical thickness < 2 mm, or cysts located in weight-bearing bones with impending structural compromise [19]. Exclusion criteria included secondary or recurrent UBCs, pathologic fracture requiring stabilization, patients lost to follow-up within 1 year after surgery were excluded.

All procedures were performed by one of two senior orthopedic surgeons with specialized training in both bone tumor and endoscopic minimally invasive techniques. The surgical procedure adhered to a standardized four-phase protocol. First, the cyst dimensions and portal sites were identified using preoperative imaging (Supplementary Fig. 1A). The portals were positioned at convenient sites to provide a wide endoscopic field of vision and facilitate both debridement and graft delivery. Two hollow needles were then percutaneously inserted to access the cyst, establishing the working and viewing channels (Supplementary Fig. 1B). Second, after aspirating the cyst contents, both endoscopic and fluoroscopic evaluation provided a panoramic assessment of the cavity (Supplementary Figs. 2 and 3). The working cannula size was selected according to the diameter of the arthroscope. Endoscopic visualization was achieved with a 30° arthroscope, most commonly 4 mm in diameter. In smaller patients or when space was limited, a 2.7 mm 30° arthroscope was used, with the corresponding smaller cannula. Debridement was performed with specially designed angled curettes to remove granulation tissue, fibrous septa, and the cyst lining, while motorized shavers ensured efficient bulk clearance (Supplementary Video 1–2). Debris was continuously suctioned and irrigated with normal saline (Supplementary Video 3). Bleeding was managed by a combination of continuous saline irrigation, tamponade through the working cannula, and, when necessary, the use of a plasma electrode to achieve focal hemostasis. Debridement was considered complete when uniformly sclerotic bone was visualized throughout the cavity, confirmed endoscopically and fluoroscopically. Third, sclerotherapy was administered following mechanical clearance to prevent recurrence from residual active lining. Through the working portal, 3% polidocanol (approximately 1 ml polidocanol per 1 cm^3^ volume of the lesion) was instilled into the cyst cavity and allowed to dwell for over 1 min. The cavity was then copiously irrigated with normal saline to remove residual sclerosant and minimize potential side effects. Finally, bone grafting was performed via syringe through the working portal using an injectable bone substitute (Wright Medical Technology, Inc, Arlington, TN), selected according to cyst size and location. Fluoroscopic confirmation was obtained to ensure the bone graft material adequately filled the cyst defect before wound closure (Supplementary Figs. 1C and 1D).

Postoperative management followed a standardized protocol emphasizing early mobilization. Patients were placed in removable splints or slings for comfort, with range of motion exercises initiated on postoperative day one. A progressive weight-bearing protocol was implemented, beginning with partial weight-bearing at two weeks and advancing to full weight-bearing by six weeks for lower extremity lesions. Upper extremity patients progressed to full activity by four weeks postoperatively. Patients received initial follow-up through in-person clinic visits, which were scheduled after surgery. Standard radiographic image evaluations were routinely obtained during all postoperative examinations. Bone cyst consolidation at the final evaluation was classified based on Neer radiographic classification system [20, 21]. To assess functional status, Musculoskeletal Tumor Society Score (MSTS) system was evaluated at last follow-up [22].

## Results

The study cohort comprised 33 patients meeting inclusion criteria, with a male predominance (24 males, 9 females), reflecting the known epidemiology of UBCs [23]. Patient age ranged from 4–20 years, with a mean age of 10.1 years at time of surgery. The cysts were located in the femur (n = 14), humerus (n = 12), calcaneus (n = 4), ilium (n = 2), and ulna (n = 1), with an average lesion diameter of 6.6 cm. Fourteen cysts were on the left side, while 19 were on the right. Postoperative hospital stays averaged 1.2 days. The mean follow-up duration was 48.4 ± 15.1 months (range 12–76 months) (Table 1).

**Table 1:** Characteristics of the patients

Parameters	Patients (N = 33)
Age (years)	10.1 (4 to 20)
Male sex	24 (72.7%)
Site	
Femur	14
Humerus	12
Calcaneus	4
Ilium	2
Ulna	1
Mean diameter of lesions (cm)	6.6
Mean postoperative hospital stay (days)	1.2
Mean follow-up duration (months)	48.4
Healing rate	90.9%

**Fig. 1:**
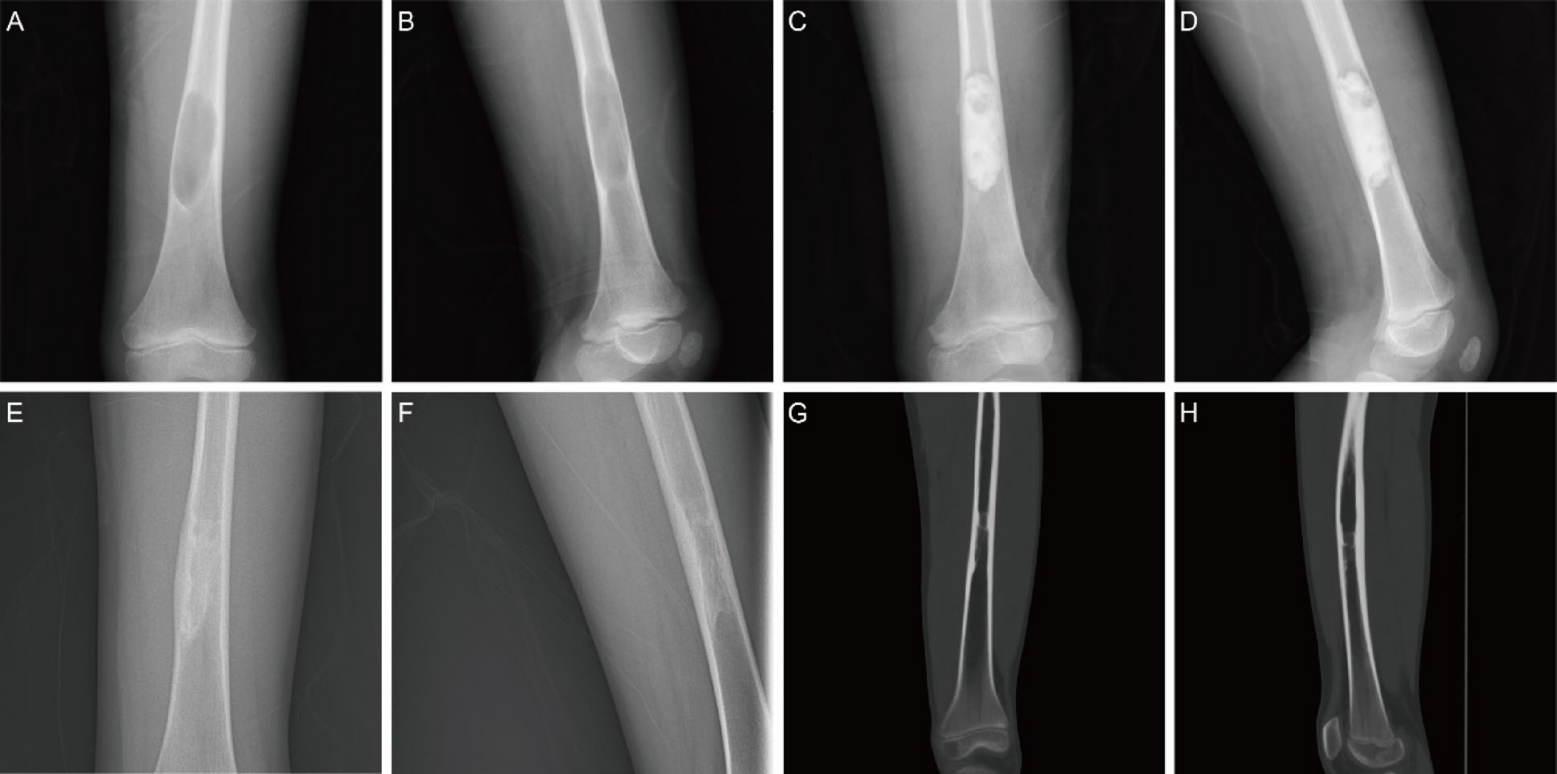
**A**, **B** Preoperative X-ray images of the femur UBC in a 6-year-old boy. **C**, **D** Postoperative X-ray images on the day after surgery. **E**, **F** Postoperative X-ray images at 28 months of follow-up. **G**, **H** Postoperative CT images at 73 months of follow-up showed complete radiographic healing

**Fig. 2:**
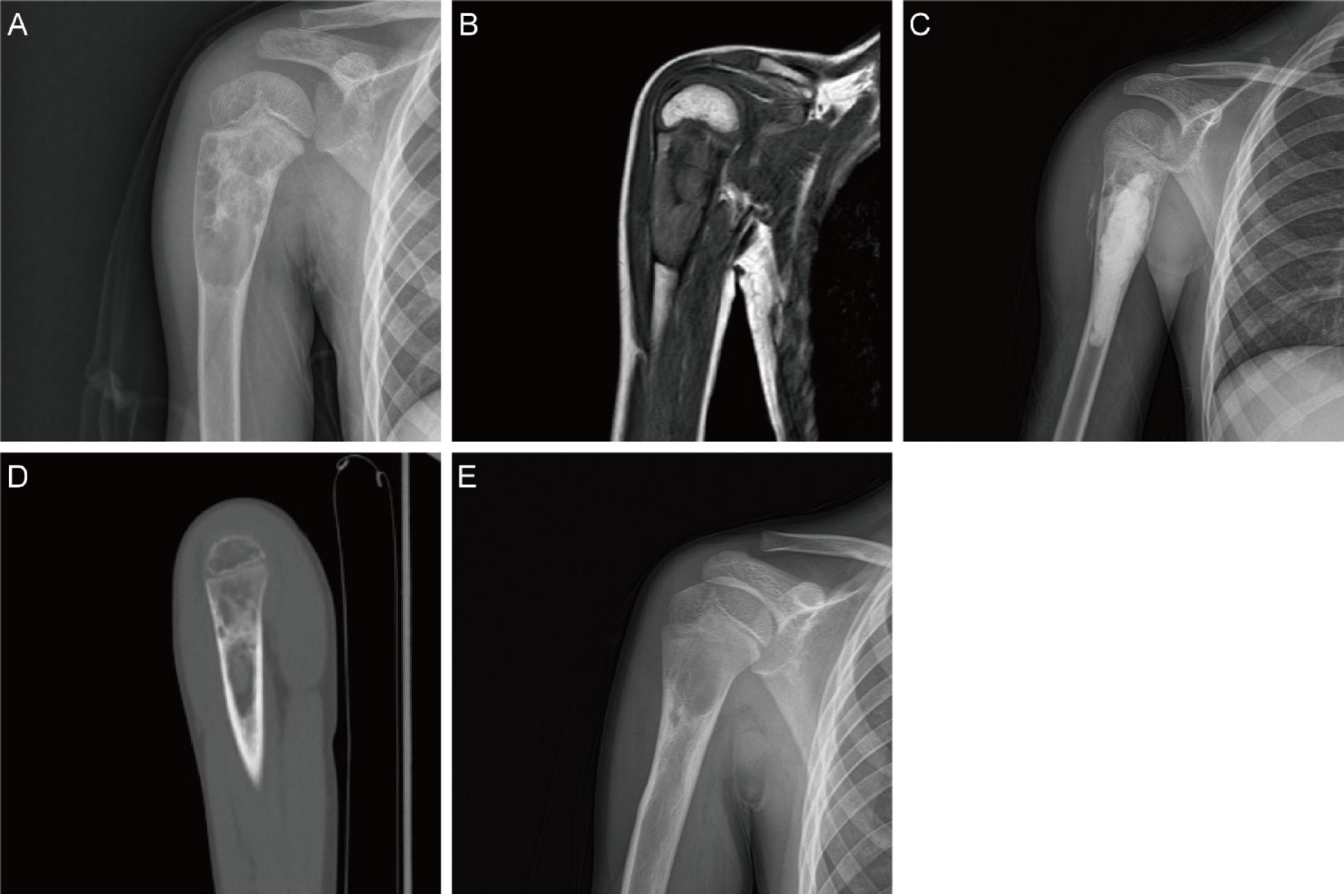
**A**, **B** Preoperative X-ray and MRI images of the humerus UBC in a 9-year-old boy. **C** Postoperative X-ray images on the day after surgery. **D** Postoperative CT images at 12 months of follow-up. **E** Postoperative X-ray images at 30 months of follow-up showed partial healing

Treatment outcomes demonstrated the efficacy of our technique. According to the modified Neer classification, complete radiographic healing or partial healing was achieved in 30 cases (90.9%), all with excellent function scores (MSTS > 28) (Figs. 1, 2); 3 cases developed persistent or recurrent cyst (9.1%) during follow-up. Among the three patients with recurrent or persistent cysts, one underwent open curettage and bone grafting and achieved complete radiographic healing. A second patient was managed with repeat endoscopic curettage and polidocanol sclerotherapy, with subsequent healing. The third patient remained asymptomatic and was managed conservatively with periodic radiographic follow-up, without evidence of cyst enlargement or fracture during the observation period.

Growth plate assessment in the 30 skeletally immature patients revealed no cases of physeal disturbance attributable to the surgical procedure. Serial radiographs indicated no angular deformities or limb length discrepancies were observed during follow-up, confirming the safety of this technique in growing children. Complications included 1 case of transient pain and 1 case of mild swelling, resolved with conservative management. No deep infections, neurovascular injuries, or pathological fractures occurred during the follow-up period. Cosmetic outcomes were excellent in all cases, with nearly imperceptible scars at surgery portal sites.

## Discussion

This study demonstrates that minimally invasive endoscopic curettage combined with polidocanol sclerotherapy and bone grafting is a safe and effective technique for the treatment of unicameral bone cysts (UBCs) in children and adolescents. Our healing rate of 90.9% is noninferior to those reported for conventional open curettage, which range from 70 to 90% in the literature [24, 25]. These results also align with previous reports of minimally invasive or endoscopic techniques for benign bone lesions, which reported healing rates of 88.9–92.3% in calcaneal cysts [26, 27]. Although recurrence occurred in 9.1% of patients, it was effectively managed either with repeat endoscopic surgery or with open curettage in one case, while an asymptomatic patient was safely observed. These findings suggest that recurrent UBCs after minimally invasive endoscopy remain amenable to secondary interventions and do not preclude ultimate cyst healing. Importantly, the ability to repeat the minimally invasive procedure without added morbidity represents a clinical advantage over traditional open approaches.

The superiority of our approach appears to stem from several key technical advantages. The endoscope provides high-definition magnification, enabling direct visualization and precise debridement of cyst walls and fibrous septa, thus avoiding the blind curettage inherent in percutaneous injections or traditional drilling techniques [28]. Histological studies have identified residual cyst membrane as a major cause of recurrence, and our systematic removal of all visible lining tissue, followed by sclerotherapy with polidocanol, addresses both macroscopic and microscopic disease. The adjuvant use of polidocanol, supported by its documented efficacy in aneurysmal bone cysts, likely enhances lesion eradication, particularly in blind areas inaccessible to instruments [2].

Since UBCs are often located near the growth plate, patients with open epiphyseal plates may be at risk of growth disturbances [1]. Minimally invasive endoscopy also minimizes soft tissue disruption, reduces intraoperative blood loss, and preserves the periosteum and adjacent physis—critical in skeletally immature patients to prevent growth plate injury [28]. In our series, no patient experienced growth disturbance, contrasting with the 7–100% physeal injury rate reported for open techniques [29, 30]. Preservation of periosteal blood supply likely accelerated graft incorporation, facilitating faster healing and allowing return to unrestricted activity.

The choice of graft material was tailored to defect size and location, with injectable calcium phosphate cement used for larger cavities and cancellous allograft chips for smaller defects, optimizing both structural support and osteoconductive potential. This approach, also described by other studies, not only restores immediate mechanical stability but also reduces the risk of postoperative pathological fractures, a complication absent in our cohort [18, 31].

The addition of polidocanol sclerotherapy in our protocol was deliberate. Although curettage alone can achieve satisfactory outcomes in unicameral bone cysts, recurrence rates remain significant due to residual microscopic cyst lining that is difficult to eradicate mechanically [32]. Histopathological studies suggest that residual fibrous tissue and endothelial-like lining cells play a central role in cyst persistence [5]. By chemically ablating these remnants, sclerotherapy complements mechanical curettage and enhances the likelihood of complete resolution. Previous reports on the use of sclerotherapy in both unicameral and aneurysmal bone cysts support its efficacy and safety [33, 34]. Importantly, we applied sclerotherapy uniformly in all cases rather than selectively. This decision reflects our aim to standardize the procedure, minimize variability, and maximize recurrence prevention across the cohort. Nonetheless, no local or systemic complications occurred in our series, potential risks of polidocanol should be acknowledged. Reported adverse events include local necrosis, ulceration, and pain, as well as rare systemic complications such as hypersensitivity, pulmonary embolic events, and cardiopulmonary compromise [17, 35]. These risks are uncommon when small intralesional doses are used with immediate irrigation, but they highlight the need for careful dosing, close monitoring, and preparedness for emergency intervention.

Our outcomes also confirm the cosmetic and functional advantages of endoscopy. Small, percutaneous portals produce minimal scarring—an important consideration for pediatric patients and their families, and shorter hospitalization combined with earlier return to normal activities offers tangible cost savings [36].

While our findings are promising, several limitations should be acknowledged. Its single-center, single-arm design and small sample size restrict generalizability and preclude direct comparisons with other treatments. Nonetheless, the consistency of our findings with prior studies supports their validity, though larger multicenter trials with control groups are needed for confirmation. Additionally, while polidocanol has shown reliable sclerosing effects in aneurysmal bone cysts, its role in unicameral bone cysts remains uncertain, and caution is warranted when extrapolating evidence between these biologically distinct entities. Our conclusion regarding physeal preservation was based solely on radiographic follow-up; although no angular deformity or limb length discrepancy was observed, systematic long-term functional and growth assessments were not performed. Finally, the procedure has a learning curve and requires surgeons to be proficient in both tumor surgery and endoscopic techniques. Moreover, the higher costs of equipment and bone substitutes compared with open curettage may limit its accessibility in resource-constrained settings.

## Conclusion

Endoscopic minimally invasive surgery combined with sclerotherapy and injectable bone grafting represents a safe and effective option for the management of UBCs in children and adolescents. While our results demonstrate high healing rates and low complication risks, limitations include the relatively small sample size, single-center design, and the cost of specialized equipment. Larger multicenter studies are warranted to validate these findings and assess cost-effectiveness. Despite these limitations, our protocol offers a valuable alternative to open surgery, with reduced morbidity, faster recovery, and superior cosmetic outcomes.

## Supplementary Information

Below is the link to the electronic supplementary material.

Supplementary Material 1

Supplementary Material 2

Supplementary Material 3

Supplementary Material 4

## Abbreviations

UBC: Unicameral bone cyst
MSTS: Musculoskeletal Tumor Society Scoring System

## References

[1] Cohen J. Etiology of simple bone cyst. J Bone Joint Surg. 1970;52(7):1493–7. 5472904

[2] Campanacci M, Capanna R, Picci P. Unicameral and aneurysmal bone cysts. Clin Orthop Relat Res. 1986;204:25–36. 3956013

[3] Lin AJ, Siddiqui AA, Fan B, Bennett JT, Illingworth KD, Andras LM, et al. Treatments and sequelae of pediatric pathologic proximal femur fractures due to benign bone cyst. J Pediatr Orthop. 2022;42(6):e661–6. 35667055 10.1097/BPO.0000000000002128

[4] Cha SM, Shin HD, Kim KC, Kang DH. Flexible intramedullary nailing in simple bone cysts of the proximal humerus: prospective study for high-risk cases of pathologic fracture. J Pediatr Orthop B. 2013;22(5):475–80. 23443143 10.1097/BPB.0b013e32835ec6ad

[5] Noordin S, Allana S, Umer M, Jamil M, Hilal K, Uddin N. Unicameral bone cysts: current concepts. Ann Med Surg. 2018;34:43–9. 10.1016/j.amsu.2018.06.005 PMC6138978 30224948

[6] Zhao JG, Wang J, Huang WJ, Zhang P, Ding N, Shang J. Interventions for treating simple bone cysts in the long bones of children. Cochrane Database Syst Rev. 2017;2(2):Cd010847. 28158933 10.1002/14651858.CD010847.pub3 PMC6464391

[7] Scaglietti O, Marchetti PG, Bartolozzi P. The effects of methylprednisolone acetate in the treatment of bone cysts. Results of three years follow-up. J Bone Joint Surg Br. 1979;61-b(2):200–4. 438272 10.1302/0301-620X.61B2.438272

[8] D’Amato RD, Memeo A, Fusini F, Panuccio E, Peretti G. Treatment of simple bone cyst with bone marrow concentrate and equine-derived demineralized bone matrix injection versus methylprednisolone acetate injections: a retrospective comparative study. Acta Orthop Traumatol Turc. 2020;54(1):49–58. 32175897 10.5152/j.aott.2020.01.371 PMC7243701

[9] Zhang P, Zhu N, Du L, Zheng J, Hu S, Xu B. Treatment of simple bone cysts of the humerus by intramedullary nailing and steroid injection. BMC Musculoskelet Disord. 2020;21(1):70. 32019514 10.1186/s12891-020-3054-6 PMC7001273

[10] Pala E, Trovarelli G, Angelini A, Cerchiaro MC, Ruggieri P. Modern treatment of unicameral and aneurysmatic bone cysts. EFORT Open Rev. 2024;9(5):387–92. 38726993 10.1530/EOR-24-0027 PMC11099581

[11] Ruiz-Arellanos K, Larios F, Inchaustegui ML, Gonzalez MR, Pretell-Mazzini J. Treatment and outcomes of 4,973 unicameral bone cysts: a systematic review and meta-analysis. JBJS Rev. 2024;12(1):e23. 10.2106/JBJS.RVW.23.00159 38181108

[12] Eastwood F, Raheman F, Al-Dairy G, Popescu M, Henney C, Hunwick L, et al. Healing smarter: a systematic review and meta-analysis of bioresorbable implants for paediatric forearm fractures. J Child Orthop. 2025;19(5):422–31. 40852198 10.1177/18632521251350854 PMC12364838

[13] Rafaqat W, Ahmad T, Ibrahim MT, Kumar S, Bluman EM, Khan KS. Is minimally invasive orthopedic surgery safer than open? A systematic review of systematic reviews. Int J Surg (Lond Engl). 2022;101:106616. 10.1016/j.ijsu.2022.106616 35427798

[14] Mack MJ. Minimally invasive and robotic surgery. JAMA. 2001;285(5):568–72. 11176860 10.1001/jama.285.5.568

[15] Aiba H, Kobayashi M, Waguri-Nagaya Y, Goto H, Mizutani J, Yamada S, et al. Treatment of aneurysmal bone cysts using endoscopic curettage. BMC Musculoskelet Disord. 2018;19(1):268. 30053808 10.1186/s12891-018-2176-6 PMC6064064

[16] Smolle MA, Jud L, Scheurer FA, Hoch A, Ackermann J, Fritz B, et al. Conventional vs. endoscopic-assisted curettage of benign bone tumours. An experimental study. J Orthop Surg Res. 2024;19(1):392. 38970099 10.1186/s13018-024-04859-w PMC11225110

[17] Puri A, Hegde P, Gulia A, Parikh M. Primary aneurysmal bone cysts. Bone Joint J. 2020;102-b(2):186–90. 32009434 10.1302/0301-620X.102B2.BJJ-2019-1083.R1

[18] Urban RM, Turner TM, Hall DJ, Inoue N, Gitelis S. Increased bone formation using calcium sulfate-calcium phosphate composite graft. Clin Orthop Relat Res. 2007;459:110–7. 17415007 10.1097/BLO.0b013e318059b902

[19] Döring K, Sturz GD, Hobusch G, Puchner S, Windhager R, Chiari C. Open surgical treatment of unicameral bone cysts : A retrospective data analysis. Wien Klin Wochenschr. 2024;136(19–20):547–55. 37650964 10.1007/s00508-023-02267-4 PMC11464551

[20] Neer CS, Francis KC, Marcove RC, Terz J, Carbonara PN. Treatment of unicameral bone cyst. A follow-up study of one hundred seventy-five cases. J Bone Joint Surg Am. 1966;48(4):731–45. 15580740

[21] Hou HY, Wu K, Wang CT, Chang SM, Lin WH, Yang RS. Treatment of unicameral bone cyst: a comparative study of selected techniques. The Journal of Bone and Joint Surgery-American Volume. 2010;92(4):855–62. 20360508 10.2106/JBJS.I.00607

[22] Enneking WF, Dunham W, Gebhardt MC, Malawar M, Pritchard DJ. A system for the functional evaluation of reconstructive procedures after surgical treatment of tumors of the musculoskeletal system. Clin Orthop Relat Res. 1993;286:241–6. 8425352

[23] Choi JH, Ro JY. The 2020 WHO classification of tumors of bone: an updated review. Adv Anat Pathol. 2021;28(3):119–38. 33480599 10.1097/PAP.0000000000000293

[24] Capanna R, Albisinni U, Caroli GC, Campanacci M. Contrast examination as a prognostic factor in the treatment of solitary bone cyst by cortisone injection. Skeletal Radiol. 1984;12(2):97–102. 6484606 10.1007/BF00360813

[25] Cha SM, Shin HD, Kim KC, Park JW. Does fracture affect the healing time or frequency of recurrence in a simple bone cyst of the proximal femur? Clin Orthop Relat Res. 2014;472(10):3166–76. 25002216 10.1007/s11999-014-3768-6 PMC4160469

[26] Yildirim C, Akmaz I, Sahin O, Keklikci K. Simple calcaneal bone cysts: a pilot study comparing open versus endoscopic curettage and grafting. J Bone Joint Surg Br. 2011;93(12):1626–31. 22161925 10.1302/0301-620X.93B12.27315

[27] Saraph V, Zwick EB, Maizen C, Schneider F, Linhart WE. Treatment of unicameral calcaneal bone cysts in children: review of literature and results using a cannulated screw for continuous decompression of the cyst. J Pediatr Orthop. 2004;24(5):568–73. 15308909

[28] Yildirim C, Mahiroğullari M, Kuşkucu M, Akmaz I, Keklikci K. Treatment of a unicameral bone cyst of calcaneus with endoscopic curettage and percutaneous filling with corticocancellous allograft. J Foot Ankle Surg. 2010;49(1):93–7. 20123299 10.1053/j.jfas.2009.08.005

[29] Li J, Rai S, Ze R, Tang X, Liu R, Hong P. Pediatric physeal slide-traction plate fixation for pathological distal femoral fracture caused by unicameral bone cyst in adolescents. BMC Musculoskelet Disord. 2020;21(1):503. 32727439 10.1186/s12891-020-03526-5 PMC7391518

[30] Huang C, Lü XM, Fu G, Yang Z. Chondroblastoma in the children treated with intralesional curettage and bone grafting: outcomes and risk factors for local recurrence. Orthop Surg. 2021;13(7):2102–10. 34599644 10.1111/os.13153 PMC8528993

[31] Fillingham YA, Lenart BA, Gitelis S. Function after injection of benign bone lesions with a bioceramic. Clin Orthop Relat Res. 2012;470(7):2014–20. 22290129 10.1007/s11999-012-2251-5 PMC3369099

[32] Neer CS, Francis KC, Johnston AD, Kiernan HA Jr. Current concepts on the treatment of solitary unicameral bone cyst. Clin Orthop Relat Res. 1973;97:40–51. 10.1097/00003086-197311000-00008 4590225

[33] Mascard E, Gomez-Brouchet A, Lambot K. Bone cysts: unicameral and aneurysmal bone cyst. Orthop Traumatol Surg Res. 2015;101(1 Suppl):S119-127. 25579825 10.1016/j.otsr.2014.06.031

[34] Rajeswaran S, Wiese M, Baker J, Chesterton J, Samet J, Green J, et al. Treatment of unicameral bone cysts utilizing the Sclerograft™ technique. Cardiovasc Intervent Radiol. 2024;47(3):346–53. 38409561 10.1007/s00270-024-03671-7

[35] Brosjö O, Pechon P, Hesla A, Tsagozis P, Bauer H. Sclerotherapy with polidocanol for treatment of aneurysmal bone cysts. Acta Orthop. 2013;84(5):502–5. 24171682 10.3109/17453674.2013.850013 PMC3822137

[36] Xiao C, Zhang S, Gao Z, Wang L, Dai Y, Li J. Arthroscopic management of juxta-articular proximal tibial chondroblastoma: a case report and literature review. Orthop Surg. 2025;17(1):295–309. 39508114 10.1111/os.14287 PMC11735375

