# Supplementary material for: Minimally invasive endoscopic treatment for unicameral bone cysts: technique and outcomes in a series of 33 pediatric and adolescent patients
Source: J Orthop Surg Res. 2025 Dec 18;21:45. doi: 10.1186/s13018-025-06557-7 (PMC12828951; doi:10.1186/s13018-025-06557-7)

**Supplementary Figure 1: Preoperative and intraoperative imaging**


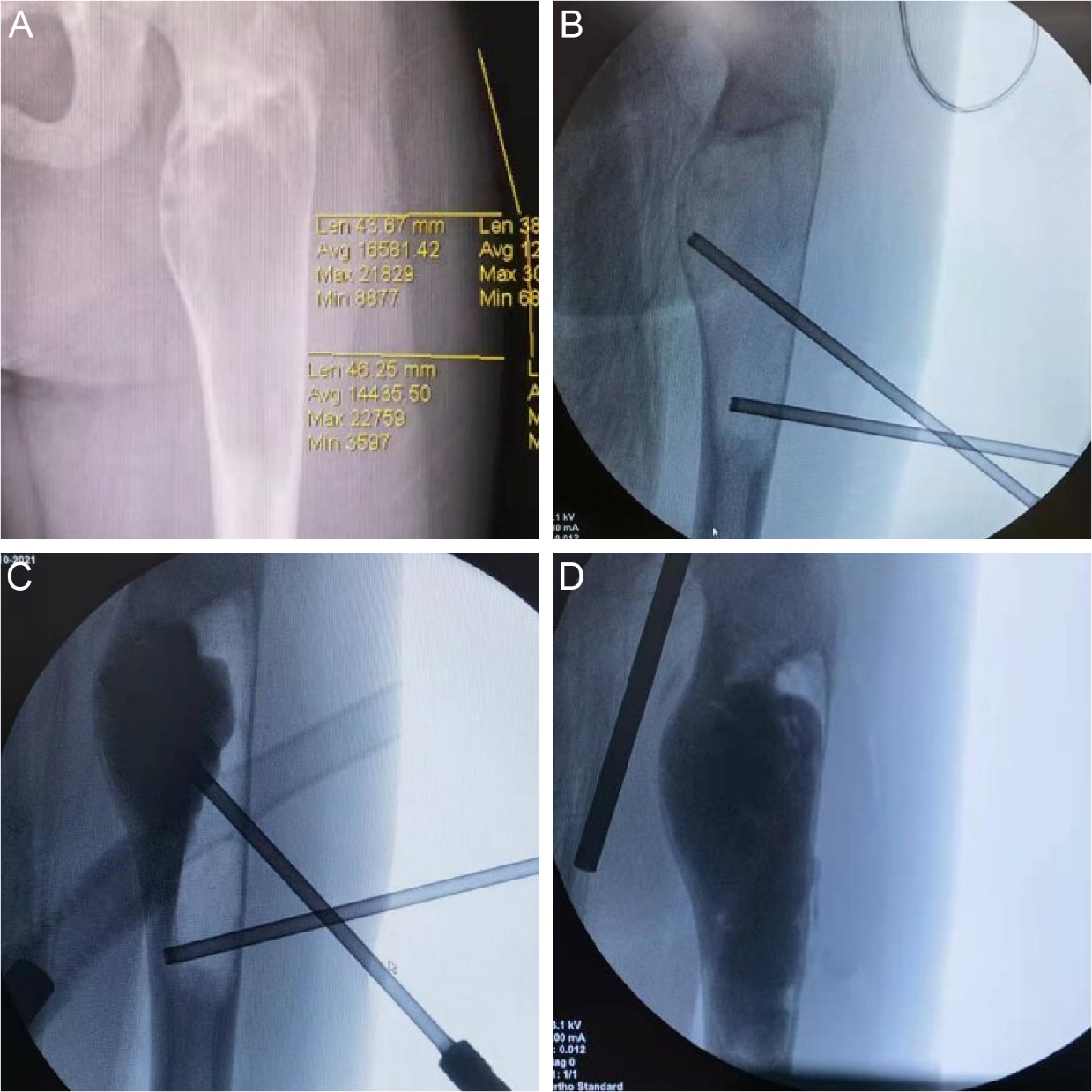


**Supplementary Figure 2:** **Endoscopic procedures under direct vision.**


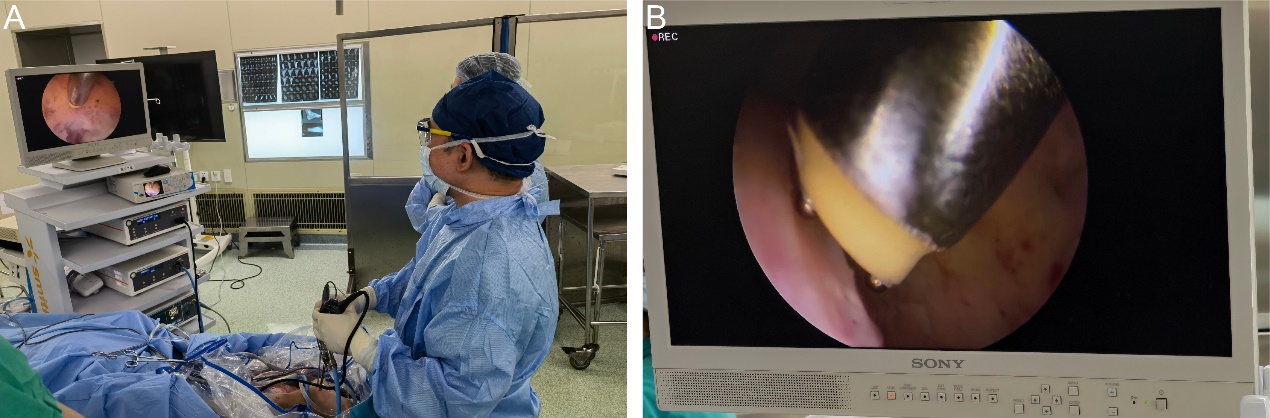

Supplement: Supplementary file 1 — Supplementary Material 1 [file 13018_2025_6557_MOESM1_ESM.docx]
